# Taxonomic and Thematic Organisation of Proper Name Conceptual Knowledge

**DOI:** 10.3233/BEN-2011-0337

**Published:** 2011-11-07

**Authors:** Sebastian J. Crutch, Elizabeth K. Warrington

**Affiliations:** Dementia Research CentreDepartment of NeurodegenerationUCL Institute of NeurologyUniversity College LondonLondonUK

**Keywords:** Semantics, conceptual knowledge, proper noun, refractoriness

## Abstract

We report the investigation of the organisation of proper names in two aphasic patients (NBC and FBI). The performance of both patients on spoken word to written word matching tasks was inconsistent, affected by presentation rate and semantic relatedness of the competing responses, all hallmarks of a refractory semantic access dysphasia. In a series of experiments we explored the semantic relatedness effects within their proper name vocabulary, including brand names and person names. First we demonstrated the interaction between very fine grain organisation and personal experience, with one patient with a special interest in the cinema demonstrating higher error rates when identifying the names of actors working in a similar film genre (e.g. action movies: Arnold Schwarzenegger, Bruce Willis, Sylvester Stallone, Mel Gibson) than those working in different genres (e.g. Arnold Schwarzenegger, Gregory Peck, Robin Williams, Gene Kelly). Second we compared directly two potential principles of semantic organisation – taxonomic and thematic. Furthermore we considered these principles of organisation in the context of the individuals' personal knowledge base. We selected topics matching the interests and experience of each patient, namely cinema and literature (NBC) and naval history (FBI). The stimulus items were arranged in taxonomic arrays (e.g. Jane Austen, Emily Bronte, Agatha Christie), thematic arrays (e.g. Jane Austen, Pride and Prejudice, Mr Darcy), and unrelated arrays (e.g. Jane Austen, Wuthering Heights, Hercule Poirot). We documented that different patterns of taxonomic and thematic organisation were constrained by whether the individual has limited knowledge, moderate knowledge or detailed knowledge of a particular vocabulary. It is suggested that moderate proper name knowledge is primarily organised by taxonomy whereas extensive experience results in a more detailed knowledge base in which theme is a powerful organising principle.

## 1. Introduction

Semantic refractory access dysphasia is a form of comprehension impairment in which access to stored conceptual knowledge is compromised. This type of access disorder is typically characterised by response inconsistency, sensitivity to temporal factors (presentation rate), sensitivity to semantic relatedness, and insensitivity to item frequency. Refractoriness is considered to be a form of neural interference which leads to "reduction in the ability to utilise the system for a period of time following activation" (Warrington and McCarthy, 1983, p. 874). For the purposes of the current paper, the critical aspect of the disorder is the sensitivity to semantic relatedness. In semantic refractory access patients not only do individual, repeatedly probed items become inaccessible but also semantically related items. Thus response accuracy is significantly lower when identifying targets which are semantically related (e.g. furniture: chair, table, stool, desk, sofa, television) than semantically unrelated (e.g. chair, pencil, door, hat, foot, telephone).

The original investigations of refractoriness were conducted using spoken word to picture matching tasks in the domain of common nouns. The build up of interference in these repetitive comprehension tasks was shown to be caused by the semantic relatedness but not the phonological relatedness of the items in the response array. For example, when provided with the spoken name 'goat', refractoriness can lead to greater

ISSN 0953-4180/11/$27.50 © 2011 – IOS Press and the authors. All rights reserved difficulty pointing to a picture of a goat presented with pictures of other semantically related items (e.g. goat, deer, bear) than with other phonologically related items (e.g. goat, boat, coat [6,20]). Refractory effects have also been explored using spoken word to written words matching tests. This technique can be used to measure semantic relatedness in patients with impaired phonological-orthographic transcoding. These matching procedures have been employed to investigate semantic relatedness in an abstract word vocabulary [7, 9] and in a proper noun vocabulary [5,6,9]. For example, it has been shown that both brand names and person names can become refractory when probed repeatedly (e.g. higher error rates for the target 'Ford' when presented in written arrays of semantically related [i.e. Ford, Vauxhall, Rover, Honda] than unrelated items [Ford, Tesco, Cadburys, Hitachi]). Measurements of semantic relatedness effect sizes can be taken as a marker of the similarity of a set of items, and thus be used to assess the validity of hypothetical semantic distinctions. Consequently one of the main motivations for studying patients with semantic refractory access dysphasia is that they offer a window on the fine-grain organisation of conceptual knowledge [19].

In our previous investigations of semantic refractory access disorders, we have focussed upon two issues: (a) Evidence for fine-grain organisation within the semantic system, and (b) The different principles which underpin this organisation. One area of enquiry which unites these two issues is the investigation of geographical names [5,9]. It has been shown that refractory patients find it more difficult to comprehend the names of geographical terms (e.g. countries, cities, counties) when selecting a response from an array of geographically close rather than geographically distant places. The influence of geographical location and proximity revealed evidence of very fine grain organisation in the patients' knowledge of geographical names; geographical proximity effects persisted even amongst English cities and London suburbs. Furthermore, these proximity effects were shown to cross taxonomic boundaries. For example, geographically close town, region and river names (e.g. London, Surrey, Thames) elicited higher error rates that names drawn from the same taxonomic class (e.g. Thames, Loire, Rhine). These Experiments suggest that at least two principles (taxonomic and geographic proximity) govern the organisation of our understanding of geographical names.

In this paper we explore a principle other than taxonomy which guides the organisation of other classes of proper names. For the purpose of these experiments, this additional principle of organisation is broadly labelled 'thematic'. This distinction between taxonomy and theme has been anticipated in a number of previous studies [10,15,16] and a number of terms have been used to capture such different forms of semantic relationship (e.g. similarity-contiguity, paradigm-syntagm; Coltheart, 1981). It is well recognised that our conceptual system allows for the representation of different forms of semantic relationship such as category membership [1], and associative connections based on subject or expert report [12,14]. Indeed it has also been suggested that concrete and abstract words may differ in their dependence upon similarity-based and associative information [7]. Barsalou and colleagues suggest that information is clustered more by theme than taxonomy for abstract words (and vice versa for concrete concepts) and argue that thematic and taxonomic clustering reflects a drive to collect concepts into groups which support goal-directed action [22].

A third issue central to our investigation of semantic refractory access disorders is the influence of personal experience upon the organisation of conceptual knowledge. In the geographical experiments described above, the probing of geographical names was predicated upon the assumption that patients had knowledge of their position within a spatial framework. This assumption was tested by comparing their comprehension of domestic and foreign place names. For example, for the English patients tested, geographical relatedness effects were observed among UK city names but not among US state names. We have argued that personal knowledge and experience interact with the semantic relatedness effects observed in this class of patient.

In the current study, our aim was to examine whether different principles of semantic organisation could be directly compared and contrasted in a variety of proper noun vocabularies. It was hypothesised that, within the realms of personal knowledge, more powerful forms of semantic relatedness than mere taxonomic class might emerge. This hypothesis was tested by developing stimulus sets which corresponded to the specific interests and experience of a particular patient (e.g. cinema, naval history). In the patient into whose particular interests the stimuli were designed to tap, it was anticipated that thematic relatedness would elicit greater semantic interference (as measured by high error rate) than mere taxonomic relatedness. By contrast, in the patient who did not share that particular interest, it was predicted that semantic interference would be limited to taxonomically related stimuli or not occur at all.

Table 1
The number and percentage correct responses on the background screening tests

| | | NBC | FBI |
|---|---|---|---|
| *WASI Matrix Reasoning* | | 9/32 ($T = 34$) | 10/32 ($T = 38$) |
| *Camden Pictorial Memory Test* | | 14/30 (< 5th %ile) | 30/30 (> 10th %ile) |
| *Digit span* | | 0 digits forwards | 2 digits forwards |
| *Repetition* | | 0/90 | 61/90 |
| *Reading (from Brown and Ure, 1969)* | | 0/72 | 25/72 |
| *Graded Nonword Reading Test* | | 0/20 | 0/20 |
| *Spoken nonword-written nonword match* | | 11/20 | 3/10 |
| *British Picture Vocabulary Scale (short)* | | 10/32 | 20/32 |
| *McKenna and Warrington (1978)* | | | |
| Naming | Animals | 2/10 (20%) | 6/10 (60%) |
| | Man-made artefacts | 0/10 (0%) | 2/10 (20%) |
| | Colours | 1/10 (10%) | 5/10 (50%) |
| | Body part | 1/10 (10%) | 1/10 (10%) |
| | Countries | 1/10 (10%) | 7/10 (70%) |
| | *TOTAL* | *5/50 (10%)* | *21/50 (42%)* |
| Comprehension | Animals | 8/10 (80%) | 10/10 (100%) |
| | Man-made artefacts | 10/10 (100%) | 7/10 (70%) |
| | Colours | 6/10 (60%) | 7/10 (70%) |
| | Body part | 2/10 (20%) | 1/10 (10%) |
| | Countries | 10/10 (100%) | 10/10 (100%) |
| | *TOTAL* | *36/50 (72%)* | *35/50 (70%)* |
| *Semantic refractory access dysphasia screening tests* | | | |
| Presentation rate | Fast (1s RSI) | 103/128 (80%) | 104/128 (81%) |
| | Slow (10s RSI) | 117/128 (91%) | 121/128 (95%) |
| Semantic relatedness | Related | 100/128 (78%) | 106/128 (83%) |
| | Unrelated | 120/128 (94%) | 119/128 (93%) |
| Frequency | Low frequency | 20/32 (63%) | 29/32 (91%) |
| | Middle frequency | 21/32 (66%) | 27/32 (84%) |
| | High frequency | 22/32 (69%) | 30/32 (94%) |

## 2. Participants

NBC is a 64-year-old retired accountant who became globally aphasic following a left middle cerebral artery stroke in 2007. MRI confirmed a left MCA infarct and a left cerebellar infarct. This patient has been reported in a two previous studies (Crutch and Warrington, 2010; Crutch and Jackson, in press). NBC exhibited a very keen interest in film and cinema, and continued to attend film showings and peruse magazines and newspaper articles about famous actors and actresses.

FBI is a 70-year-old, right-handed retired sailor and building maintenance engineer who became globally aphasic following a large left middle cerebral artery ischaemic stroke in March 2005. A computed tomography (CT) scan conducted on the day of admission revealed a left parietal infarct with minimal oedema and mass effect. No further imaging is available. Information about this patient may be found in several previous single case and case series reports [8,9]. FBI was severely physically disabled by his stroke but expressed particular enthusiasm for documentaries and books about naval matters.

These patients were included in this study because they both showed (a) the characteristic features of semantic refractory access dysphasia, and (b) impaired phonological-orthographic transcoding so that spoken word to written word matching tasks could be employed as a measure of semantic processing (see below for confirmatory details of the patient's neuropsychological profiles).

## 3. Background information

NBC completed a short battery of tests of neuropsychological function (see Table 1). He showed low average-borderline non-verbal reasoning performance on the Matrix Reasoning subtest from the Wechsler Abbreviated Scale of Intelligence [21]. However he appeared unable to understand the Camden Pictorial Memory Test [18]. Phonological deficits were apparent in his failure to read any items from the Graded Nonword Reading test [17], and his chance score when required to point to a specified cvc given a choice of two nonwords with no letters in common (e.g. fip – dag). He was also completely unable to repeat or read a single word aloud including single digits. NBC demonstrated a severe anomia, managing to produce the names of very few of the high frequency items from McKenna and Warrington [13]. He obtained a weak score on the short form of the British Picture Vocabulary Scale [11], and showed impaired performance on a simple spoken word-picture test involving high frequency items [4, 13]. In a series of spoken word to picture matching tasks, NBC's comprehension performance was shown to be (a) sensitive to presentation rate, (b) sensitive to semantic relatedness, but (c) insensitive to item frequency (see Table 1).

FBI completed the same short battery of tests of neuropsychological function as NBC (see Table 1). He showed low average-borderline non-verbal reasoning performance on the Matrix Reasoning subtest from the WASI. Visual episodic memory was well preserved as indicated by strong performance on the Camden Pictorial Memory Test. However, short term memory as measured by digit span was severely impaired. FBI was able to read and repeat a large proportion of the single words presented (see Warrington and Crutch, 2007, for full details of FBI's dyslexia). Phonological skills were severely compromised as he failed to read any items from the Graded Nonword Reading test, and scored at chance when required to point to a specified cvc given a choice of two nonwords with no letters in common (e.g. fip – dag). Naming was also severely compromised, with FBI producing the names of less than half of the high frequency items from McKenna and Warrington [13]. On tests of semantic processing, he obtained a weak score on the BPVS, and this comprehension deficit was mirrored by impaired performance on the simple spoken word-picture tests involving high frequency items. On spoken word to picture matching tasks, FBI was also shown to be sensitive to presentation rate and semantic relatedness but not item frequency (see Table 1).

The neuropsychological profiles indicate that both patients exhibited global aphasia. The combination of the hallmark features of semantic refractory access dyslexia and these patients' impaired phonological-orthographic transcoding meant that it was therefore appropriate to administer repetitive spoken word to written word matching tasks as a measure of semantic processing.

## 4. Experimental investigation

All of the experiments reported in this investigation employ a spoken word to written word matching procedure, in which the patients were presented with an array of 3 or 4 written words and then the examiner spoke the name of each word aloud several times in a fixed random order, on each occasion requiring the patient to point to the word they had just heard. As noted above, this procedure was an appropriate means of investigating the patients' comprehension of these words because their abolished nonword reading and spoken-nonword-to-written-nonword matching performance indicated minimal residual capacity for phonological processing of the written word. Thus any accurate word reading and spoken-written matching was held to reflect semantic processing.

### *Experiment 1 – Semantic relatedness effects in brand names and person names*

Before comparing directly the influence of taxonomic and thematic relatedness in multiple proper noun domains, we examined whether the semantic relatedness effects observed previously in NBC and FBI's comprehension of place names were also observed in the proper noun domains of brand names and people. These tests constitute a replication (Experiment 1a) and extension (Experiment 1b) of previous single case evidence of proper noun relatedness effects [6]. In particular, patient NBC had reported a particular interest in cinema, so a novel task involving famous film actors was administered.

*Methods*

The comprehension of two sets of proper nouns were examined:

a) Brand names – these test materials were previously described in Crutch and Warrington (2004; Experiment 3). The stimuli consisted of 16 well-known brand names, four drawn from each of the categories of supermarkets, car manufacturers, food producers and electrical goods manufacturers. These were arranged into four semantically close arrays (e.g. Safeway, Tesco, Waitrose, and Asda) and four semantically distant arrays (e.g. Safeway, Rover, Kelloggs, and Panasonic).
b) Person names (cinema) – the names of 16 famous actors were probed under two relatedness conditions. Semantically related arrays involved the names of four actors who work primarily in a similar cinematic genre (e.g. action movies: Arnold Schwarzenegger, Bruce Willis, Sylvester Stallone, Mel Gibson). Semantically unrelated arrays involved the same names rearranged so that each array included one actor from each different genre (e.g. Arnold Schwarzenegger, Gregory Peck, Robin Williams, Gene Kelly).

All stimuli are shown in the Appendix. The two sets of stimuli were administered on separate occasions, and all arrays were presented in an ABBA design with an approximate 1s responses stimulus interval.

*Results*

The number and percentage correct responses on each task in Experiment 1 are shown in Table 2. Performance in the related and unrelated conditions was evaluated using Wilcoxon matched-pairs signed-ranks tests. On the brand names task, both patients showed significant semantic relatedness effects, responding less accurately with related than unrelated brand names. On the specialised person task involving film actors, only NBC who was particularly interested in cinema showed a significant relatedness effect.

*Comment*

These results suggest that the comprehension performance of both patients on spoken word to written word matching tasks involving proper noun domains other than geographical terms was sensitive to semantic relatedness. More specifically, as is typical of patients with semantic refractory access dysphasia, response accuracy was reduced in conditions of semantic relatedness, indicating greater semantic interference among the item representations. These data replicate and extend those reported previously for another semantic refractory access dysphasic patient, AZ [6].

*Experiment 2 – Direct comparisons of taxonomic and thematic relatedness*

In the second section of the investigation, semantic relatedness effects were exploited to directly compare and contrast the influence of two different types of information upon item comprehension: taxonomic class and thematic association[1]. As noted in the Introduction, sensitivity to semantic relatedness is constrained by the amount of knowledge available (e.g. through personal experience, education). Here we explore the impact of personal knowledge upon word comprehension across several domains of conceptual knowledge.

[1] It should be noted that taxonomic class and thematic association are not mutually exclusive, as items within a taxonomic class often co-occur in our linguistic or real world experience. By reference to other terms used in the aphasic literature, the essential distinction of interest here is between similarity/shared features and co-occurrence/association.

*Methods*

The influence of taxonomic and thematic relatedness upon comprehension performance (relative to an unrelated control condition) was examined in 4 different vocabularies. Two of these domains of knowledge were selected to match NBC's main interests (literature and film), and 2 were selected to match FBI's main interests (naval history; see Appendix). In all cases, the overall experimental design was identical. Stimuli ($N = 9$ or 16) were tested under three conditions – taxonomic, thematic, and unrelated – with conditions varied in an ABCABC or Latin square design. The domains of knowledge examined were:

a) Literature – the stimuli were authors, novels and characters, arranged as follows:
   - Taxonomy: 3 authors (e.g. Jane Austen, Emily Bronte, Agatha Christie), 3 novels and 3 characters
   - Thematic: Related author-novel-character groupings (e.g. Jane Austen, Pride and Prejudice, Mr Darcy)
   - Unrelated: Unrelated author-novel-character groupings (e.g. Jane Austen, Wuthering Heights, Hercule Poirot)
b) Film – the stimuli were the names of films and their respective directors, actors and actresses, arranged as follows:
   - Taxonomy: 4 films (e.g. Chinatown, Rear Window, Taxi Driver, Some Like it Hot), 4 directors, 4 actors and 4 actresses
   - Thematic: Related film-director-actor-actress groupings (e.g. Chinatown, Roman Polanski, Jack Nicholson, Faye Dunaway)
   - Unrelated: Unrelated film-director-actor-actress groupings (e.g. Chinatown, Billy Wilder, Robert De Niro, Grace Kelly)
c) Naval explorers – the stimuli were the names famous explorers, their corresponding vessels, and the locations with which they are most associated, arranged as follows:
   - Taxonomy: 3 explorers (e.g. Ernest Shackleton, Christopher Columbus, Captain Bligh), 3 vessels and 3 locations
   - Thematic: Related explorer-vessel-location groupings (e.g. Ernest Shackleton, Endurance, Antarctica)
   - Unrelated: Unrelated explorer-vessel-location groupings (e.g. Ernest Shackleton, Santa Maria, Tahiti)

Table 2
Number and percentage correct responses in each related and unrelated condition in Experiment 1

| | | | Related | Unrelated | Related vs unrelated |
|---|---|---|---|---|---|
| 1a | Brands | NBC | 34/64 | 59/64 | 3.19 |
| | | | (53%) | (92%) | $P = 0.001$ |
| | | FBI | 33/64 | 57/64 | 3.41 |
| | | | (52%) | (89%) | $P = 0.0007$ |
| 1b | Actors | NBC | 47/64 | 58/64 | 2.04 |
| | | | (73%) | (91%) | $P = 0.04$ |
| | | FBI | 37/64 | 45/64 | 0.89 |
| | | | (58%) | (70%) | $P > 0.3$ |

Table 3
Number and percentage correct responses in each taxonomic, thematic and unrelated condition in Experiment 2. Wilcoxon matched-pairs signed-ranks statistical pairwise comparisons of taxonomic, thematic and unrelated conditions

| | | | Taxonomic | Thematic | Unrelated | Taxonomic vs thematic | Taxonomic vs unrelated | Thematic vs unrelated |
|---|---|---|---|---|---|---|---|---|
| 2a | Literature | NBC | 35/36 | 26/36 | 35/36 | −2.14 | 0.00 | 2.39 |
| | | | (97%) | (72%) | (97%) | $P = 0.03$ | $P = 1$ | $P = 0.02$ |
| | | FBI | 23/36 | 35/36 | 30/36 | 2.19 | 1.51 | −1.73 |
| | | | (64%) | (97%) | (83%) | $P = 0.03$ | $P = 0.13$ | $P = 0.08$ |
| 2b | Film | NBC | 56/64 | 42/64 | 58/64 | −2.36 | 0.18 | 3.09 |
| | | | (88%) | (66%) | (91%) | $P = 0.02$ | $P = 0.86$ | $P = 0.002$ |
| | | FBI | 46/64 | 57/64 | 54/64 | 2.22 | 1.52 | −1.00 |
| | | | (72%) | (89%) | (84%) | $P = 0.03$ | $P = 0.13$ | $P = 0.32$ |
| 2c | Explorers | NBC | 32/36 | 21/36 | 29/36 | −2.21 | −1.34 | 1.81 |
| | | | (89%) | (58%) | (81%) | $P = 0.03$ | $P = 0.18$ | $P = 0.07$ |
| | | FBI | 36/36 | 29/36 | 35/36 | −2.41 | −1.00 | 1.91 |
| | | | (100%) | (81%) | (97%) | $P = 0.02$ | $P = 0.32$ | $P = 0.06$ |
| 2d | Naval battles | NBC | 28/36 | 28/36 | 33/36 | −0.06 | 0.93 | 1.04 |
| | | | (78%) | (78%) | (92%) | $P = 0.95$ | $P = 0.35$ | $P = 0.30$ |
| | | FBI | 18/36 | 33/36 | 28/36 | 2.27 | 2.39 | −1.45 |
| | | | (50%) | (92%) | (78%) | $P = 0.02$ | $P = 0.02$ | $P = 0.15$ |

d) Naval battles – the stimuli were the names of renowned naval battles and the commanders and vessels most associated with them, arranged as follows:

- Taxonomy: 3 naval battles (e.g. Jutland, Matapan, Falklands), 3 commanders and 3 vessels
- Thematic: Related battle-commander-vessel gr- oupings (e.g. Jutland, Jellicoe, Indefatiguable)
- Unrelated: Unrelated battle-commander-vessel groupings (e.g. Jutland, Cunningham, Belgrano)

*Results*

The number and percentage correct response on Experiments 2a-d are shown in Table 3 and Fig. 1. In considering the results, it should be noted that, paradoxically, higher error rates are considered to indicate a higher degree of knowledge of the stimuli being tested (and hence a greater susceptibility to the build up of semantic interference). The corollary is that lower error rates are considered to indicate a lesser degree of knowledge of the stimuli (and hence lower susceptibility to semantic interference). The unrelated arrays condition provides an estimate of baseline word knowledge.

*Experiments 2a - Literature*

On the first set of stimuli selected to match NBC – his interest in and knowledge of literature – NBC's performance was most impaired in the thematically related condition as compared with both the taxonomic and unrelated conditions. For example, his error rate was much higher when asked simply to point to a thematically associated triplet of names (e.g. Jane Austen, Pride and Prejudice, Mr Darcy) than a taxonomically related triplet of the names of three authors (e.g. Jane Austen, Emily Bronte, Agatha Christie). This is despite the many common features of the taxonomic items (e.g. gender, profession, etc.). This was also observed in the context of his baseline performance of 97% correct in the unrelated condition.

By contrast, FBI showed a quite different pattern of performance across the three conditions, with the high-

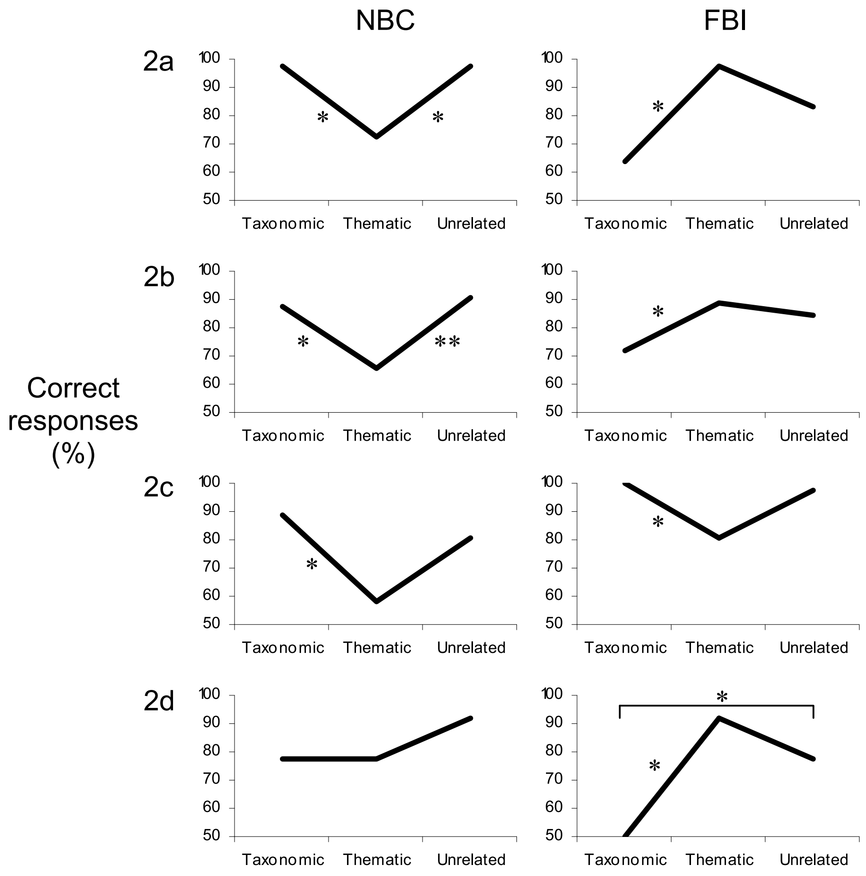

Fig. 1. The percentage correct responses in Experiment 2.

est error rate in the taxonomically related condition. There was a trend toward the opposite pattern to NBC, namely a higher error rate on the taxonomically related as compared to thematically related condition.

*Experiment 2b – Film*

On the second set of stimuli selected to suit NBC – his keen interest in film and cinema – NBC's performance was again most impaired in the thematically related condition. He showed significantly higher error rate in the thematically related condition (e.g. Rear Window, Alfred Hitchcock, James Stewart, Grace Kelly) than either the taxonomically related (e.g. Roman Polanski, Alfred Hitchcock, Martin Scorsese, Billy Wilder) or unrelated conditions. However there were no comparable differences between his performances on the taxonomically related and unrelated conditions.

Patient FBI was apparently insensitive to these dimensions, scoring at a satisfactory level on all three conditions and with no significant differences between either the thematic or taxonomic conditions relative to the unrelated condition.

*Experiments 2c – Explorers*

The third set of stimuli was selected to match FBI's areas of personal interest and knowledge in naval history. On the Explorers task (Experiment 2c), both NBC and FBI showed a mild thematic effect. Both showed statistical trends toward lower scores on the thematic condition (e.g. Christopher Columbus, America, Santa Maria) than the control unrelated condition in both patients, but there were no differences between performances on the taxonomically related (e.g. Christopher Columbus, Ernest Shackleton, Captain Bligh) and unrelated conditions.

*Experiment 2d – Naval battles*

On the more demanding Naval battles task, NBC showed no pairwise differences between conditions. However, FBI showed significantly lower performance on the taxonomic (e.g. Matapan, Jutland, Falklands) than unrelated condition. There was no accompanying difference between the thematic (e.g. Jellicoe, Jutland, Indefatiguable) and unrelated conditions.

*Comment*

Patient NBC showed significant thematic effects in 3/4 Experiments, including both the tasks designed to focus on his particular areas of knowledge and interest (literature and cinema) and also one of the tasks designed with patient FBI in mind (explorers). By contrast patient FBI showed no thematic or taxonomic effects on tasks examining literature or cinema, though by his adequate performance in the unrelated conditions demonstrated these items were clearly within his vocabulary. However, FBI did show a thematic effect with explorers, and perhaps more impressively a significant taxonomic effect with naval battles, indicating familiarity with this obscure topic.

## 5. Discussion

In this study we have explored aspects of proper name knowledge hitherto neglected. In particular, we examined the principles and extent of fine-grain organisation within this conceptual domain, and also considered the influence of personal experience upon comprehension performance.

In Experiment 1, we reported data indicating the fine-grain organisation of both brand names and person names in two semantic refractory access dysphasic patients, NBC and FBI. For brand names, semantic refractoriness was greater (yielding higher error rates) for related (e.g. Safeway, Tesco, Waitrose, Asda) than unrelated (e.g. Safeway, Rover, Kelloggs, Panasonic) items. This performance replicated that of patient AZ [6]. The impact of personal experience upon the extent of fine grain organisation was then explored using stimuli drawn from the area of expertise of one patient (NBC) but not the other (FBI), namely male film actors associated with a particular cinematic genre. Patient NBC showed significantly higher errors rates when identifying the names of actors who worked in a similar genre (e.g. action movies: Arnold Schwarzenegger, Bruce Willis, Sylvester Stallone, Mel Gibson) than those who worked in different genres (e.g. Arnold Schwarzenegger, Gregory Peck, Robin Williams, Gene Kelly). Patient FBI was relatively insensitive to this manipulation. This pattern of refractory performance indicates fine grain organisation of person knowledge not just between occupations (as previously documented in Crutch and Warrington [6]) but even within a single occupation. The caveat here is that the absolute level of fine grain organisation is dictated to a large extent by personal experience and other individual factors.

In Experiment 2, we compared directly two potential principles of semantic organisation – taxonomy and theme – across domains of knowledge in which our two patients had varied levels of expertise. In the areas of literature and cinema (Experiments 2a and 2b), NBC showed greater refractoriness (shown by significantly higher error rates) in the thematic condition than in the taxonomic and unrelated conditions. Of particular interest is the observation in Experiment 2a that associated concepts (e.g. Jane Austen, Pride and Prejudice, Mr Darcy) generated more semantic interference than taxonomically similar concepts, despite the fact that the latter share many more semantic features (e.g. Jane Austen, Emily Bronte, Agatha Christie – all female, all novelists, all deceased, etc.). By contrast, FBI showed higher error rates in the taxonomic condition, suggesting that he had moderate but not as detailed knowledge as NBC about these authors, novels and characters. This suggests that within NBC's conceptual knowledge base, thematically-related items (e.g. authors, novels, characters) are represented more closely in semantic space than taxonomically-related items (e.g. individuals from the category of authors).

In areas of particular personal knowledge (naval history) for FBI, both patients in fact showed significantly more semantic interference in the thematic condition of the Explorers task (Experiment 2c). It should be noted thought that the stories of all three of the explorers selected are relatively well-known and have been made into mainstream films, which might account for why not only FBI's but also NBC's performance indicated familiarity with the specific associations between different explorers, ships and destinations. On the more demanding and obscure naval battles test however, it was only FBI who showed any significant differences in performance between conditions. He showed greater semantic interference in the taxonomic than thematic or unrelated conditions, suggesting he possessed strong knowledge for the battle, ship and commander names used but perhaps did not retain adequate knowledge to be clear on the exact thematic connections. NBC showed no significant evidence of semantic relatedness effects on this task, suggesting weak knowledge of this topic.

Three broad patterns of performance emerged from Experiment 2. For the purposes of this Discussion we label these patterns 'limited knowledge', 'moderate knowledge' and 'detailed knowledge', and these are illustrated schematically in Fig. 2. 'Limited knowledge',

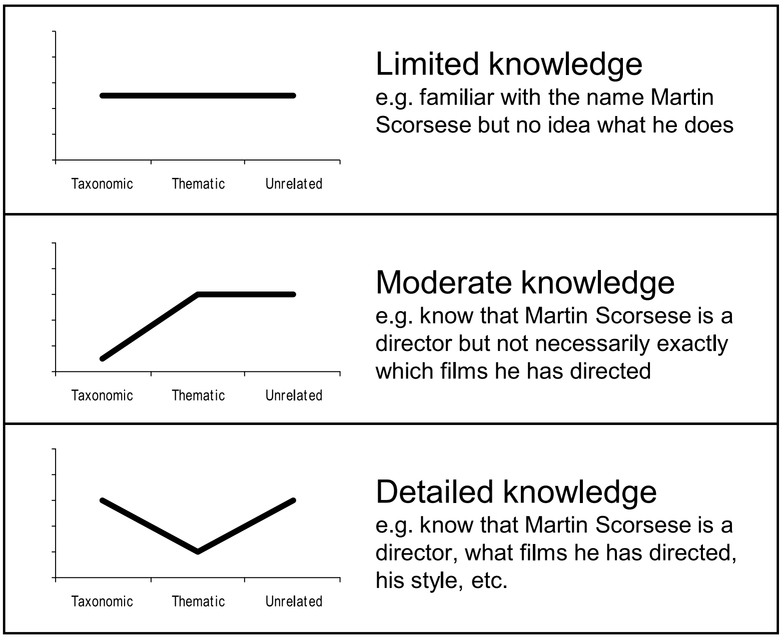

Fig. 2. Hypothetical patterns of performance across the taxonomic, thematic and unrelated conditions for different degrees of personal knowledge.

as demonstrated by NBC on task 2d, indicates that the verbal labels are within one's vocabulary but little more detail is available (e.g. familiar with the name Jellicoe but no idea what he does). As a consequence, no significant difference is observed between related and unrelated arrays. 'Moderate knowledge', as demonstrated by FBI on tasks 2a, 2b and 2d, indicates enough knowledge has been acquired to achieve a taxonomic classification of the term but insufficient detail to have individual thematic associations (e.g. know that Martin Scorsese is a film director but not which individual films he is associated with). As a consequence, taxonomic arrays yield a significantly higher error rate than thematic and/or unrelated arrays. 'Detailed knowledge', as demonstrated by NBC on tasks 2a-c and FBI on task 2c, indicates that individual thematic associations between concepts have been acquired (e.g. know that Martin Scorsese is a film director and that he directed Taxi Driver). In this instance, a v-shaped pattern is observed in which thematic arrays yield a significantly higher error rate than taxonomic or unrelated arrays.

The counterintuitive aspect of this pattern of performance is that there may be no significant difference between taxonomically related and unrelated items. This absence of a taxonomic effect does not mean that information about taxonomic class has been lost, but rather that the taxonomic effect has been over-taken by a more powerful thematic effect. An analogy could be drawn here to the organisation within small and large libraries: in a very small library, Agatha Christie and Jane Austen novels might both be found on the 'Fiction' bookcase, whereas in a larger library they might be found on separate bookcases for 'Crime' and 'Classics'. Thus a more detailed classification can increase the distance between items which are taxonomically related. In the human brain, this organisation of psychological space is mirrored in neural organisation, with the representations or patterns of activation underpinning taxonomically related concepts becoming more distinct with increasing knowledge. This increase in taxonomic semantic distance with increasing experience is a feature of the computational models of Rogers and McClelland (2004). By training networks to be 'experts' in either bird or fish knowledge by manipulating the exposure during the training phase, these researchers demonstrated a tendency to name at the subordinate level in the expert domain and at the basic level in the non-expert domain. The greater differentiation of subordinate items in the expert domain was also demonstrated by greater mean Euclidean distances between the networks internal representations.

A few further points regarding patient performance and experimental design are worth noting. First, the experimental approach described is dependent on patients having sufficiently impaired phonological-orthographic transcoding as to permit spoken word to written word matching tasks to be used as a measure of semantic processing. Nonetheless both patients demonstrated a remarkable insensitivity to variables such as the number of words in the stimulus (e.g. pointing to 'Death on the Nile' in response to the auditory probe 'Agatha Christie'). Second, one potential limitation to this line of investigation is that the experiments were tailor-made to the patients we were studying. Not all individuals have an area of specialist knowledge or interest, and it is not possible to match or balance for the extent of this knowledge across subject areas. Nevertheless, it can be argued that single case methodology provides an opportunity to study aspects of the cerebral organisation of personal knowledge which would be difficult within a group study design.

In conclusion, many investigations of conceptual knowledge have examined a vocabulary which is familiar to the majority of individuals within a society. However, there are many aspects of personal experience and education which have a powerful influence on how this common database is extended. The current study points to the role of both taxonomic and thematic information in the fine grain organisation of proper name knowledge. These principles play a critical role in creating the personalised cerebral knowledge base through which we understand and interact with the world.

## Acknowledgements

We would like to thank Dr David Cohen and the therapists on the Harrow Stroke Unit and the Regional Rehabilitation Unit at Northwick Park Hospital for allowing us to work with patients under their care. This work was undertaken at UCLH/UCL who received a proportion of funding from the Department of Health's NIHR Biomedical Research Centres funding scheme. The Dementia Research Centre is an Alzheimer's Research Trust Co-ordinating Centre. This work was supported by an Alzheimer's Research Trust Senior Research Fellowship to SC.

## Appendix

| | | | |
|---|---|---|---|
| **Experiment 1b** | | | |
| *Semantically related* | | | |
| *Screen legends* | *Action movie actors* | *Comic actors* | *Musical/Dance actors* |
| Gregory Peck | Arnold Schwarzenegger | Robin Williams | Gene Kelly |
| James Stewart | Bruce Willis | Steve Martin | Fred Astaire |
| Humphrey Bogart | Sylvester Stallone | Jim Carrey | Mickey Rooney |
| Clark Gable | Mel Gibson | Chevy Chase | Frank Sinatra |
| *Semantically unrelated* | | | |
| Gregory Peck | James Stewart | Humphrey Bogart | Clark Gable |
| Arnold Schwarzenegger | Bruce Willis | Sylvester Stallone | Mel Gibson |
| Robin Williams | Steve Martin | Jim Carrey | Chevy Chase |
| Gene Kelly | Fred Astaire | Mickey Rooney | Frank Sinatra |
| **Experiment 2a** | | | |
| *Taxonomically related* | | | |
| *Authors* | *Novels* | *Characters* | |
| Jane Austen | Pride and Prejudice | Mr Darcy | |
| Emily Bronte | Wuthering Heights | Heathcliff | |
| Agatha Christie | Death on the Nile | Hercule Poirot | |
| *Thematically related* | | | |
| Jane Austen | Emily Bronte | Agatha Christie | |
| Pride and Prejudice | Wuthering Heights | Death on the Nile | |
| Mr Darcy | Heathcliff | Hercule Poirot | |
| *Unrelated* | | | |
| Jane Austen | Death on the Nile | Pride and Prejudice | |
| Wuthering Heights | Emily Bronte | Heathcliff | |
| Hercule Poirot | Mr Darcy | Agatha Christie | |
| **Experiment 2b** | | | |
| *Taxonomically related* | | | |
| *Films* | *Directors* | *Actors* | *Actress* |
| Chinatown | Roman Polanski | Jack Nicholson | Faye Dunaway |
| Rear Window | Alfred Hitchcock | James Stewart | Grace Kelly |
| Taxi Driver | Martin Scorsese | Robert De Niro | Jodie Foster |
| Some Like it Hot | Billy Wilder | Jack Lemmon | Marilyn Monroe |
| *Thematically related* | | | |
| Chinatown | Rear Window | Taxi Driver | Some Like it Hot |
| Roman Polanski | Alfred Hitchcock | Martin Scorsese | Billy Wilder |
| Jack Nicholson | James Stewart | Robert De Niro | Jack Lemmon |
| Faye Dunaway | Grace Kelly | Jodie Foster | Marilyn Monroe |
| *Unrelated* | | | |
| Rear Window | James Stewart | Robert De Niro | Jodie Foster |
| Martin Scorsese | Marilyn Monroe | Grace Kelly | Alfred Hitchcock |
| Jack Lemmon | Taxi Driver | Chinatown | Jack Nicholson |
| Faye Dunaway | Roman Polanski | Billy Wilder | Some Like it Hot |
| **Experiment 2c** | | | |
| *Taxonomically related* | | | |
| Vessel | Explorer/Captain | Location | |
| HMS Bounty | Captain Bligh | Tahiti | |
| Santa Maria | Christopher Columbus | America | |
| Endurance | Ernest Shackleton | Antarctica | |
| *Thematically related* | | | |
| HMS Bounty | Santa Maria | Endurance | |
| Captain Bligh | Christopher Columbus | Ernest Shackleton | |
| Tahiti | America | Antarctica | |

| | | |
|---|---|---|
| *Unrelated* | | |
| Santa Maria | Christopher Columbus | America |
| Ernest Shackleton | Antarctica | Endurance |
| Tahiti | HMS Bounty | Captain Bligh |
| **Experiment 2d** | | |
| *Taxonomically related* | | |
| *Vessel* | *Commander* | *Battle* |
| Warsprite | Cunningham | Matapan |
| Indefatiguable | Jellicoe | Jutland |
| Belgrano | Woodward | Falklands |
| *Thematically related* | | |
| Warsprite | Indefatiguable | Belgrano |
| Cunningham | Jellicoe | Woodward |
| Matapan | Jutland | Falklands |
| *Unrelated* | | |
| Belgrano | Indefatiguable | Falklands |
| Jutland | Woodward | Warsprite |
| Cunningham | Matapan | Jellicoe |
